# Comparative Investigation of the Rheological Properties and Rejuvenation Mechanism of Rejuvenated SBS Modified Asphalt Binder After Ultraviolet Aging

**DOI:** 10.3390/ma19051041

**Published:** 2026-03-09

**Authors:** Fucheng Guo, Xu He, Pengfei Zhi, Hongmei Ma, Hui Dou, Bo Li

**Affiliations:** 1 Gansu Industry Technology Center of Transportation Construction Materials Research and Application, Lanzhou Jiaotong University, Lanzhou 730070, China; fcguo@chd.edu.cn (F.G.);; 2 Gansu Provincial Transportation Research Institute Group Co., Ltd., Lanzhou 730030, China

**Keywords:** SBS modified asphalt binder, ultraviolet aging, rheological properties, rejuvenation mechanism, rejuvenators

## Abstract

This study aims to investigate the restorative effects and rejuvenation mechanisms of two rejuvenators on ultraviolet (UV)-aged SBS modified asphalt binder. Two types of rejuvenators were developed. The rheological properties of aged and rejuvenated asphalt were systematically evaluated using a dynamic shear rheometer (DSR), bending beam rheometer (BBR), and multiple stress creep and recovery (MSCR) tests. Fourier transform infrared spectroscopy (FTIR) and gel permeation chromatography (GPC) were employed to analyze the rejuvenation mechanisms. The results demonstrate that UV aging significantly deteriorates both the high- and low-temperature performance of SBS modified asphalt binder. Oil-rich rejuvenator A effectively restores UV-aged asphalt’s high-temperature performance and low-temperature stiffness. Polymer-based rejuvenator B better repairs PAV-aged cross-linked networks with superior chemical dilution, but over-dilutes large molecules. Both comparably restore aged low-temperature performance, with rejuvenator A favoring stiffness recovery and rejuvenator B favoring m-value recovery. FTIR analysis reveals that aging significantly increases the carbonyl and sulfoxide indices of SBS modified asphalt binder, especially after PAV and UV aging. Rejuvenator B exhibits superior chemical dilution, reducing these indices nearly to their original levels. GPC analysis demonstrates an aging-induced molecular weight increase and large molecular size (LMS) formation. The recovery effect of rejuvenator A is quite limited (reducing LMS by 2%). Conversely, rejuvenator B aggressively reduces LMS but causes over-dilution. Overall, rejuvenator B is recommended to be used for aged SBS modified asphalt binder, especially after UV aging.

## 1. Introduction

Styrene–Butadiene–Styrene (SBS) modified asphalt binder has been widely adopted in modern highway engineering, due to its superior resistance to high-temperature rutting, low-temperature cracking, and fatigue-induced deterioration. The incorporation of SBS polymers enhances the viscoelastic properties of asphalt binder, improving its adaptability to fluctuating temperatures and varying traffic loads. Consequently, SBS modification significantly extends the service life of asphalt pavements, particularly in regions subjected to extreme climatic variations [1,2]. However, despite these advantages, SBS modified asphalt binder remains highly susceptible to aging, primarily due to prolonged exposure to environmental stressors such as oxidation, ultraviolet radiation, moisture, and mechanical loading from traffic [3]. These aging mechanisms inevitably lead to structural degradation, reducing the long-term performance and durability of asphalt pavements.

Asphalt aging is mainly caused by thermal oxidation and UV radiation. These two factors work together and lead to a worse rheological performance. They also increase stiffness and reduce fatigue resistance. As a result, pavement cracking and rutting get worse [4,5]. SBS modified asphalt is now widely used in road engineering. Therefore, it is very important to study how different aging mechanisms affect its durability and service life. It is also key to find effective restoration methods. Short-term aging (STA) happens mainly at high temperatures and in oxygen-rich environments. During this process, light components, namely saturates and aromatics, evaporate and oxidation occurs [6]. Long-term aging usually causes polymer degradation. It also increases stiffness and brittleness [7]. Although the initial resistance to permanent deformation improves, the brittleness and fatigue cracking risks increase [8]. Besides thermal oxidation, UV radiation speeds up surface aging through photochemical oxidation, especially in the strong ultraviolet region of northwest China. It also damages the asphalt matrix and the SBS polymer network [9]. Long-term UV exposure harms the elastic structure of SBS. This greatly reduces its strengthening ability and elasticity. Consequently, there is a higher likelihood of permanent deformation occurring [10]. Also, elastic recovery capacity is seriously weakened. Therefore, it is of utmost importance to identify effective restoration methods to tackle the performance deterioration resulting from the aforementioned issues of thermal oxidation and UV aging. Against this backdrop, employing rejuvenators has emerged as a pivotal strategy for reviving the performance of aged asphalt and prolonging the lifespan of pavement.

Studies have demonstrated that rejuvenators work by replenishing the light components lost in aged asphalt, thereby restoring its elasticity and viscosity [11]. Common types include mineral oils, bio-based materials, and industrial by-products. Their mechanisms of action vary, and their effectiveness depends on both the extent of asphalt aging and the chemical composition of the rejuvenator [12]. Multiple studies have investigated the restorative effects of rejuvenators on aged asphalt. Sun et al. [13] found that mineral oil-based rejuvenators significantly improve high-temperature performance. Wang et al. [14] reported that rejuvenators reduce the softening point, complex shear modulus, and average recovery rate, while increasing the phase angle and non-recoverable creep. Liu et al. [15] demonstrated that rejuvenators decrease the creep stiffness (S) and increase the m-value through low-temperature bending creep tests, thereby enhancing the flexible deformation capacity of aged asphalt. Zhang et al. [16] further confirmed that rejuvenators effectively replenish light components and partially restore the activity of functional groups. However, the existing research has primarily focused on the performance improvement of long-term aged asphalt [17]. However, studies on the rejuvenation mechanisms of SBS modified asphalt under UV aging, particularly regarding the micro-scale mechanisms post-UV exposure, remain limited.

UV aging induces severe surface deterioration in asphalt pavements through photochemical reactions, posing a significant threat to their service life. Unlike thermal oxidation, UV radiation specifically damages the polymer network in modified asphalt, leading to a sharp decline in elasticity and crack resistance. Zhu et al. [18] investigated nanocomposites with different ratios of CeO_2_/montmorillonite (MMT) and demonstrated that the synergistic effect between CeO_2_’s UV absorption capability and MMT’s layered physical barrier effectively mitigates UV aging. Ju et al. [19] found that the rutting factor of asphalt increased during the initial UV aging phase, while this increment gradually diminished with prolonged exposure. Extended exposure to high-intensity UV radiation ultimately leads to micro-crack formation on the asphalt surface, while the degraded SBS modifier further compromises asphalt homogeneity. Furthermore, Li et al. [20] experimentally confirmed that appropriately increasing the mixing temperature and duration enhances the adhesion of rejuvenated asphalt. Their self-developed rejuvenator diluted the carbonyl and sulfoxide groups in aged asphalt and effectively inhibited oxidative cross-linking of large asphalt molecules. Although rejuvenators have been demonstrated to partially restore the properties of UV-aged asphalt, their effectiveness in repairing UV-induced damage remains insufficiently explored. The current research predominantly focuses on macroscopic performance recovery, while investigations into the underlying chemical rejuvenation mechanisms are still inadequate, particularly those addressing polymer network degradation and molecular weight variations under the PAV and UV aging for SBS modified asphalt.

This study aims to systematically evaluate the effects of different rejuvenators on the macro- and micro-scale properties of SBS modified asphalt binders under UV aging. Specifically, DSR, BBR, and MSCR tests are used to characterize the rheological behavior of rejuvenated SBS modified asphalt binder after UV aging under low temperatures and high temperatures. Moreover, FTIR and GPC are employed to elucidate rejuvenation effects and underlying mechanisms at the molecular scale. Simultaneously, the results are compared with the binder after the conventional thermal-oxidative aging. The expected results provide guidance for the selection and formulation optimization of rejuvenators during the regeneration of UV-aged asphalt.

## 2. Materials and Methods

### 2.1. Raw Materials

#### 2.1.1. SBS Modified Asphalt Binder

The SBS modified asphalt binder was prepared by adding a linear SBS modifier into base asphalt binder (Zhenhai#90), which was provided by Gansu Province Transportation Planning Survey & Design Institute Co., Ltd. (Lanzhou, China) The technical indicators of the base asphalt binder were measured as shown in Table 1 [21], which meets the specification requirement (JTG E20-2011 [22]). The SBS content of 4.5% was used in this study, which has been widely verified by previous publications and engineering experience for its high cost-effectiveness [23].

#### 2.1.2. Rejuvenator A

Rejuvenator A is a bio-based rejuvenator that was independently developed by the Road Institute of Lanzhou Jiaotong University (Lanzhou, China). Rejuvenator A consists of waste vegetable oil, a plasticizer, a toughening agent, and a tackifier with a ratio of 74:15:4:7, and its compositions are shown in Table 2 [24].

#### 2.1.3. Rejuvenator B

Rejuvenator B was developed by Gansu Provincial Transportation Science Research Institute Group Co., Ltd. (Lanzhou, China). Its main components include a softener, plasticizer, polymerization agent, aluminate coupling agent, restorative, antioxidant BHT, UV absorber UV-326, and SBS. The detailed compositions are presented in Table 3.

### 2.2. Test Methods

#### 2.2.1. Research Methodology Overview

**Figure 1:**
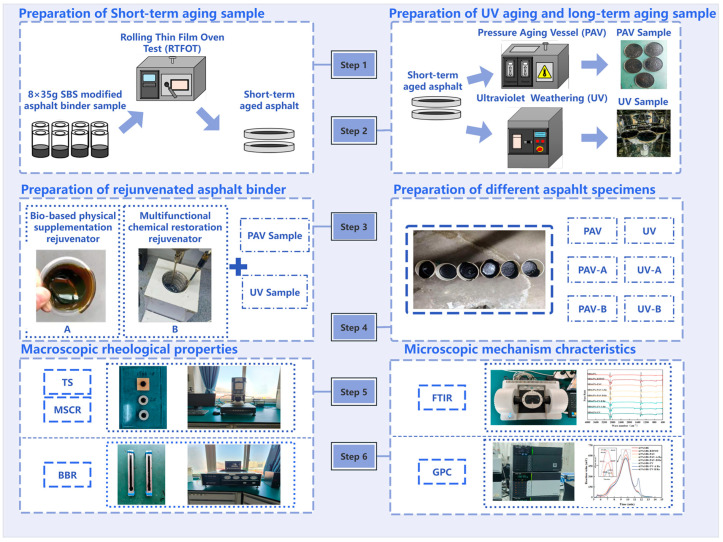
Research methodology flowchart of this study.

In this study, SBS modified asphalt binder, aged asphalt binder after short-term, long-term and UV, and rejuvenated asphalt binder with two rejuvenators were prepared, respectively. A rheological performance test and microscopic characteristic test were conducted. The comparison analysis was conducted further for different types of binder. The research methodology in this study is shown in Figure 1.

#### 2.2.2. Preparation of Aged and Rejuvenated SBS Modified Asphalt Binder

The RTFOT was employed in this study to prepare the aged asphalt binder, due to its ability to form a thin asphalt binder film and its reduced test duration, which effectively simulates the short-term aging of asphalt binder mixtures during mixing. Initially, asphalt binder samples were placed in the RTFOT and aged at 163 °C ± 0.5 °C for 85 min, yielding short-term aged asphalt binder [25]. Subsequently, the STA asphalt was further conditioned in a PAV at 105 °C and 2.1 MPa for 20 h to obtain a long-term aged asphalt binder. In parallel, a separate batch of short-term aged asphalt binder was subjected to UV aging in a dedicated chamber, where it was exposed to UV irradiation at an intensity of 800 W/m^2^ and a temperature of 50 °C for 18.6 h, resulting in UV-aged asphalt binder [26].

For rejuvenated asphalt binder, the aged SBS modified asphalt binder sample (short-term aging, long-term aging, or UV-aging) with the content of 200 g was heated to a molten state, followed by the addition of 10% by the weight of rejuvenator A or B at 170 °C directly. The mixture was then stirred thoroughly for 5 min to ensure the uniform dispersion of the rejuvenators. Thus, the regenerated SBS modified asphalt binder was produced (denoted as SBS-PAV-A/B or SBS-UV-A/B, respectively).

#### 2.2.3. Rheological Performance Test

(1) Temperature scan test.

DSR is a key instrument to evaluate the high-temperature rheological performance of the asphalt binder. The evaluation parameters, including the complex shear modulus G* and the phase angle *δ*, were obtained. The complex shear modulus is a key parameter for evaluating the viscoelastic properties of asphalt binder. It characterizes the material’s overall stiffness and deformation response under dynamic shear loading conditions. Temperature scanning tests were carried out on the unaged, aged and regenerated SBS modified asphalt binder by a dynamic rheological shear tester. The loading angular velocity was 10 rad/s, with a shear strain of 3%. The heating rate was maintained at 2 °C/min [27]. The variations in G* and *δ* and other viscoelastic parameters at different temperatures from 52 °C to 76 °C were studied. A larger G* indicates stronger resistance to shear deformation at high temperatures and a smaller *δ* indicates better recovery ability, due to a higher elastic component. In terms of composite indices, a higher G*/*sinδ* indicates stronger rutting resistance. Three replicate samples were used for the DSR test, and three replicate samples were also prepared for the following BBR tests, FTIR, and GPC analysis. The test results were averaged.

(2) Multiple stress creep recovery test.

The MSCR test was widely used to accurately evaluate the rutting resistance of the asphalt binder under high-temperature conditions. The test applies repeated loading and unloading to obtain the *J_nr_* and the percent recovery *R* at different stress levels. *J_nr_* quantifies the tendency for permanent deformation after loading. A larger *J_nr_* indicates poorer resistance to nonrecoverable deformation and greater rutting susceptibility at high temperatures. *R* measures the elastic recovery of the material after unloading. A larger *R* indicates stronger recovery and better rutting resistance at high temperatures. High-quality modified asphalt binders typically show lower *J_nr_* and higher *R*, combining strong deformation resistance with good elastic recovery under MSCR loading at a high temperature. The MSCR test was carried out on the unaged, aged and regenerated SBS modified asphalt binder. The test temperature was 52 °C to 76 °C and the heating amplitude was 6 °C. The stress control mode was adopted, and the continuous test was carried out at 0.1 and 3.2 kPa stress levels. The MSCR consists of 1 s loading, followed by 9 s recovery. The MSCR test was to evaluate the high temperature rutting resistance of the modified asphalt binder by the *R* and *J_nr_* values [28]. A higher *R* value and lower *J_nr_* value represent satisfactory high-temperature deformation resistance.

(3) Low-temperature bending creep test

The low-temperature performance is tested by BBR, and the specimen is prepared in the form of a small beam. A constant load is applied at the specified temperature. The mid-span deflection is recorded with the time. The *S* and *m* are calculated from the load–deflection relationship. The *S* indicates the ability of the asphalt binder to resist deformation at a low temperature. A higher value means higher rigidity and a higher risk of brittle cracking. The creep rate m-value shows the ability to relax stress with time at a low temperature. A higher value means better stress relaxation and better resistance to cracking. In this study, the criteria of a creep stiffness not exceeding 300 MPa and a creep rate no less than 0.3 are adopted for the evaluation of low-temperature performance. The *S* and *m* at 60 s were measured by a BBR test to reflect the low temperature performance of asphalt binder. The specimen is trabecular, with a length of 127.00 mm, a thickness of 6.35 mm and a width of 12.70 mm. The test temperatures are set as −12 °C, −18 °C, and −24 °C, respectively [29].

#### 2.2.4. Microscopic Characteristic Test

(1) Infrared spectroscopy test method.

FTIR is a widely used technique for analyzing the chemical functional groups in compounds. Different materials exhibit varying degrees of absorption to infrared radiation at different wavelengths, generating distinct infrared spectra. Specific chemical bonds or functional groups correspond to characteristic infrared absorption peaks. By analyzing the position, intensity, quantity, and shape of these peaks, changes in functional groups and molecular structures within a sample can be qualitatively or quantitatively assessed. The typical wave number range used for the FTIR analysis of materials spans from 4000 cm^−1^ to 400 cm^−1^ [30]. The peak areas of the carbonyl group at 1700 cm^−1^ and the sulfoxide group at 1030 cm^−1^ in the asphalt change significantly under the influence of aging. Therefore, the area method is commonly employed for the quantitative analysis of asphalt FTIR test data.

The data analysis was performed using the spectral analysis software OMNIC 9.2. First, the transmittance data were converted into absorbance data, followed by automatic baseline correction, automatic smoothing, and vertical axis normalization.

The peak areas at 1700 cm^−1^ and 1030 cm^−1^ were measured. Simultaneously, considering that aliphatic groups are less susceptible to aging and their peak areas remain relatively stable during the aging process, their characteristic peak areas (at approximately 1460 cm^−1^ and 1376 cm^−1^) were measured as reference peaks. The ratio of the functional group characteristic peak area to the reference peak area—defined as the carbonyl index and the sulfoxide index—serves as an indicator to quantify the impact of aging on the functional groups of asphalt [31]. The calculation formulas are as follows:

(1) $$I_{C=O}=\frac{A_{C=O}}{A_{ref}}$$

(2) $$I_{S=O}=\frac{A_{S=O}}{A_{ref}}$$

where *I_C=O_* is the carbonyl index and *I_S=O_* is the sulfoxide index.

(2) Test method for gel permeation chromatography.

The GPC is a liquid phase size exclusion chromatography technique used to separate and analyze polymers based on molecular size. In this study, 35 mg of the asphalt binder sample (original, aged, and rejuvenated) was dissolved in 2 mL of tetrahydrofuran and magnetically stirred for 12 h at room temperature to ensure complete dissolution. The solution was filtered through a 0.45 μm PTFE membrane and injected into a GPC system equipped with styrene-divinylbenzene columns at 35 °C. Tetrahydrofuran served as the mobile phase at a flow rate of 1.0 mL/min. Asphalt is primarily composed of complex hydrocarbons, in which aromatics and asphaltenes contain abundant conjugated double bonds and polycyclic aromatic hydrocarbons. Since 254 nm falls within the strong absorption bands of these molecules, significantly enhancing detection sensitivity, a UV detector (at λ = 254 nm) was employed to identify aromatic compounds and degradation products [32].

**Figure 2:**
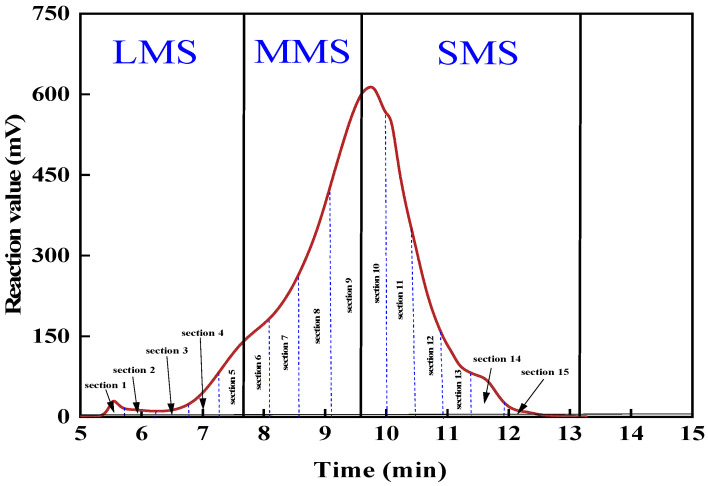
Schematic of GPC result analysis method.

Calibration was performed using polystyrene standards to determine the relative molecular weights of the sol fraction generated by the vulcanization de-crosslinking of activated rubber powder at various reaction times, thereby inferring the extent of de-crosslinking. The weight average molecular weight (*M_w_*) was calculated according to Equation (3). Furthermore, following the GPC analytical method illustrated in Figure 2, the gel permeation chromatogram profile was first divided into 15 equal-width slices. When the retention time reached the end of the 5th, 9th, and 15th slices, respectively, the large molecular size (LMS), medium molecular size (MMS), and small molecular size (SMS) were quantified as the relative areas under the curve. Based on the integral analysis of the characteristic peaks in the GPC spectra, the relative content distribution of different molecular weight components in the samples can be quantitatively characterized by calculating the percentage of the chromatographic peak areas corresponding to the high, medium, and low molecular weight regions, relative to the total peak area [33].

(3) $$M_{w}=\frac{\sum N_{i}M_{i}^{2}}{\sum N_{i}M_{i}}$$

where *M_i_* is the molecular weight and *M_w_* is the average molecular weight.

## 3. Results and Discussion

### 3.1. Rheological Properties of Different Aged and Rejuvenated Asphalt Binder

#### 3.1.1. Analysis of Temperature Scan Result

(1) Complex shear modulus.

**Figure 3:**
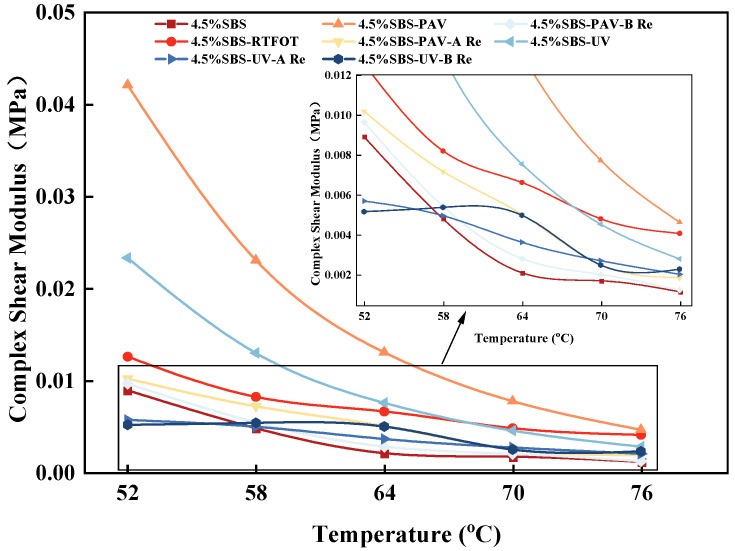
Effect of temperature on complex shear modulus for different asphalt binder.

The variations in complex shear modules of aged SBS binder with different rejuvenators is shown in Figure 3, where SBS refers to SBS modified asphalt binder. SBS-RTFOT, SBS-PAV, and SBS-UV refer to SBS modified asphalt binder after RTFOT aging, PAV aging, and UV aging, respectively. SBS-PAV-A Re and SBS-PAV-B Re refers to the rejuvenated SBS modified asphalt binder after PAV aging, with the addition of rejuvenator A and rejuvenator B. SBS-UV-A Re and SBS-UV-B Re refers to the rejuvenated SBS modified asphalt binder after UV aging, with the addition of rejuvenator A and rejuvenator B.

As shown in Figure 3, different aging types and rejuvenation treatments significantly influence the rheological properties of SBS modified asphalt binder. Specifically, the original asphalt binder maintains the lowest G* in the tested temperature range (52–76 °C), demonstrating an excellent viscoelastic balance. In comparison, PAV aging increases the modulus by 170%, whereas UV aging results in an approximate 100% increase. This discrepancy arises because PAV aging primarily forms a dense three-dimensional network through oxidative cross-linking reactions, significantly enhancing intermolecular forces and leading to a sharp rise in the modulus [34]. In contrast, although UV aging also induces polymer chain scission and oxidation, its damaging effect is limited by the penetration depth of ultraviolet radiation, which primarily affects the material surface, resulting in a relatively smaller modulus increase. Regarding rejuvenation effects, the two rejuvenators exhibit distinct restoration mechanisms for UV-aged asphalt. Samples treated with rejuvenator A show a modulus of 0.014 MPa, closer to that of the original asphalt binder, whereas samples restored with rejuvenator B maintain a higher modulus of 0.02 MPa. This difference stems from their compositional characteristics: rejuvenator A, with its high content of waste vegetable oil, effectively replenishes light components and reduces system viscosity, while rejuvenator B partially rebuilds the elastic network while softening the matrix through the inclusion of SBS polymer and restorative agents [35].

(2) Phase angle.

**Figure 4:**
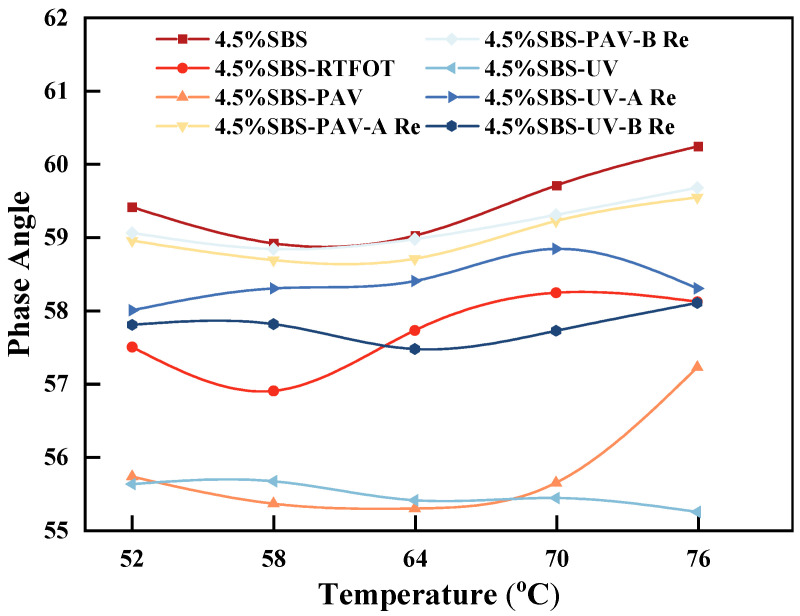
Effect of temperature on phase angle of different types of asphalt binder.

The effect of different rejuvenators on the phase angle for the aged SBS modified asphalt binder was obtained from DSR experiments, as shown in Figure 4.

As shown in Figure 4, the *δ* value of different SBS modified asphalt binders demonstrates significant temperature dependence. The *δ* values of UV-aged asphalt fluctuate within the range of 55–56°, intermediate between unaged and PAV-aged levels, indicating that photo-oxidation primarily occurs at the surface with weak temperature sensitivity. Compared with UV-aged asphalt, both UV-A Re and UV-B Re increase the *δ* value by approximately 3° at 52–58 °C. The *δ* value of UV-B Re is about 2° lower than that of UV-A Re, demonstrating that while both rejuvenators restore elasticity, rejuvenator B retains greater elasticity at high temperatures, thereby providing superior high-temperature deformation stability. At the same temperature, the *δ* of UV-B Re remains lower than that of PAV-B Re. Although rejuvenator B significantly reduces the δ of PAV-aged asphalt, residual irreversible aging under severe PAV oxidation prevents it from reaching the level achieved in UV-B Re. Similarly, the *δ* value of UV-A Re is generally slightly lower than that of PAV-A Re. This difference occurs because the oil-based composition of rejuvenator A more effectively repairs polymer degradation induced by UV radiation, whereas the crosslinked structure formed during PAV aging requires the elastic network restoration function of rejuvenator B for targeted repair.

(3) Rutting factor.

**Figure 5:**
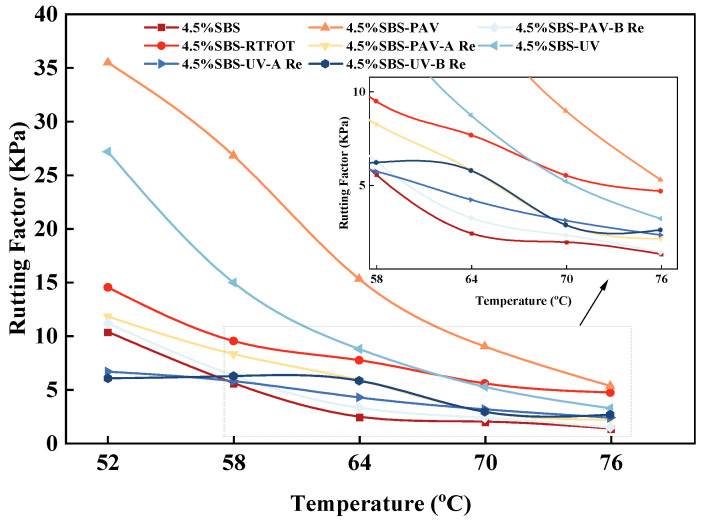
Variation in rutting factors with temperature for different types of asphalt binder.

The effect of different rejuvenators on the rutting factor for the aged SBS modified asphalt binder was also obtained from DSR experiments, as shown in Figure 5.

As shown in Figure 5, the original SBS modified asphalt binder maintains the lowest rutting factor within the 52–76 °C range, while PAV aging significantly increases it to 35 kPa, and UV aging leads to an approximate 300% increase. This difference originates from the fact that PAV aging forms a three-dimensional network structure through oxidative cross-linking, substantially enhancing stiffness, whereas UV photo-oxidative aging primarily induces surface polymer degradation due to its limited penetration depth. In terms of rejuvenation effects, rejuvenator A restores the rutting factor of UV-aged samples to a level close to that of the original asphalt binder, while samples treated with rejuvenator B still maintain a higher value. The mechanism behind this lies in rejuvenator A’s effective replenishment of light components and reduction in viscosity through its 74% vegetable oil content, whereas rejuvenator B partially preserves the elastic network while softening the matrix by incorporating SBS polymer and restorative agents. Notably, for deeply PAV-aged asphalt, rejuvenator B demonstrates superior restoration effects compared to rejuvenator A, validating the specificity of rejuvenator B’s polymer reconstruction function in repairing cross-linked networks, while rejuvenator A’s oil replenishment mechanism is more targeted toward polymer degradation caused by UV aging [36].

#### 3.1.2. Analysis of MSCR Result

The MSCR experimental method is proposed for high-temperature performance evaluation. The MSCR experiment simulates the linear and nonlinear viscoelastic responses of the asphalt binder through two stress levels (0.1 kPa and 3.2 kPa), and obtains two key indicators, the average recovery rate and the average unrecoverable creep compliance, which reflect the elastic response and permanent deformation capacity of asphalt binder, respectively. In this study, the MSCR experiment was carried out in the temperature range of stress levels of 0.1 kPa and 3.2 kPa and 52 °C to 76 °C to comprehensively evaluate the high-temperature performance of the asphalt binder. The test results are as follows [37].

(1) Average recovery rate.

**Figure 6:**
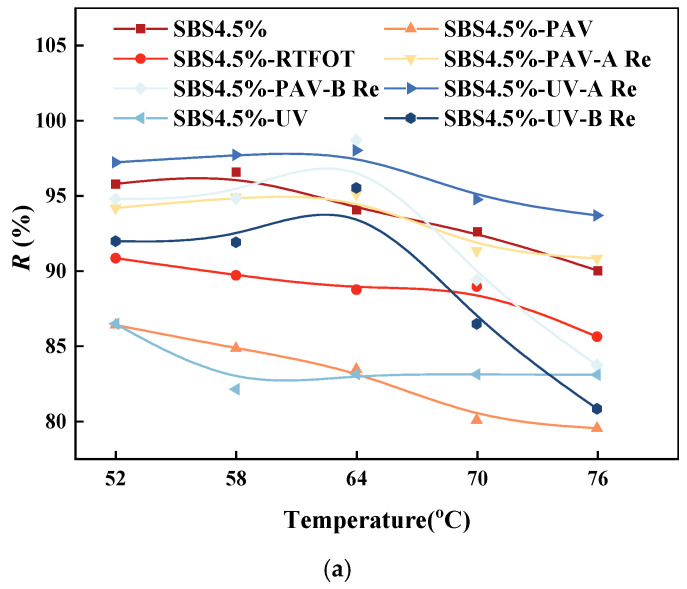

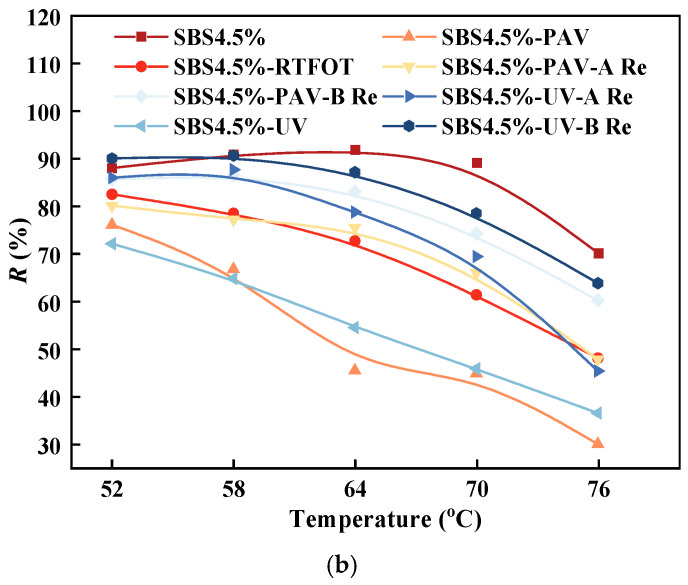
Variation in average recovery rate with temperature for different binders under a compressive stress of (**a**) 0.1 kPa and (**b**) 3.2 kPa.

The unrecoverable creep compliance for different types of asphalt binder can be obtained by MSCR, as shown in Figure 6.

As shown in Figure 6, under a stress of 0.1 kPa, the original SBS asphalt binder maintains the highest recovery rate, while aging significantly reduces this property. Specifically, PAV aging causes the *R* value to drop sharply to approximately 60%, whereas UV-aged asphalt shows a smaller decrease, remaining at around 75%. When the stress increases to 3.2 kPa, the *R* values of all samples decline, with aged samples experiencing more noticeable degradation. The *R* value of PAV-aged asphalt further decreases to about 45%. This occurs because the dense cross-linked network formed during PAV aging, while enhancing stiffness, severely restricts the movement of molecular chains, leading to a significant decline in recovery performance after stress release. Meanwhile, UV aging mainly damages the surface SBS network structure, and thus has a relatively limited impact on overall recovery. The increase in stress level exacerbates irreversible molecular chain sliding, further deteriorating recovery performance. In terms of rejuvenation effects, different rejuvenators show distinct results [38]. At 0.1 kPa stress, rejuvenator A restores the *R* value of UV-aged asphalt to approximately 82.5%, close to the original level, while rejuvenator B performs relatively weakly, with the R-value only recovering to about 66%. Notably, under 3.2 kPa high stress, the performance difference between the two rejuvenators becomes more pronounced. This distinction mainly stems from their different mechanisms: rejuvenator A effectively restores the asphalt’s fluidity and molecular chain mobility by replenishing light components, thereby significantly improving stress recovery performance. In contrast, rejuvenator B focuses on rebuilding the elastic network, which enhances stiffness but also somewhat limits the freedom of molecular chain movement, resulting in relatively limited recovery improvement [39].

(2) Irrecoverable creep compliance

**Figure 7:**
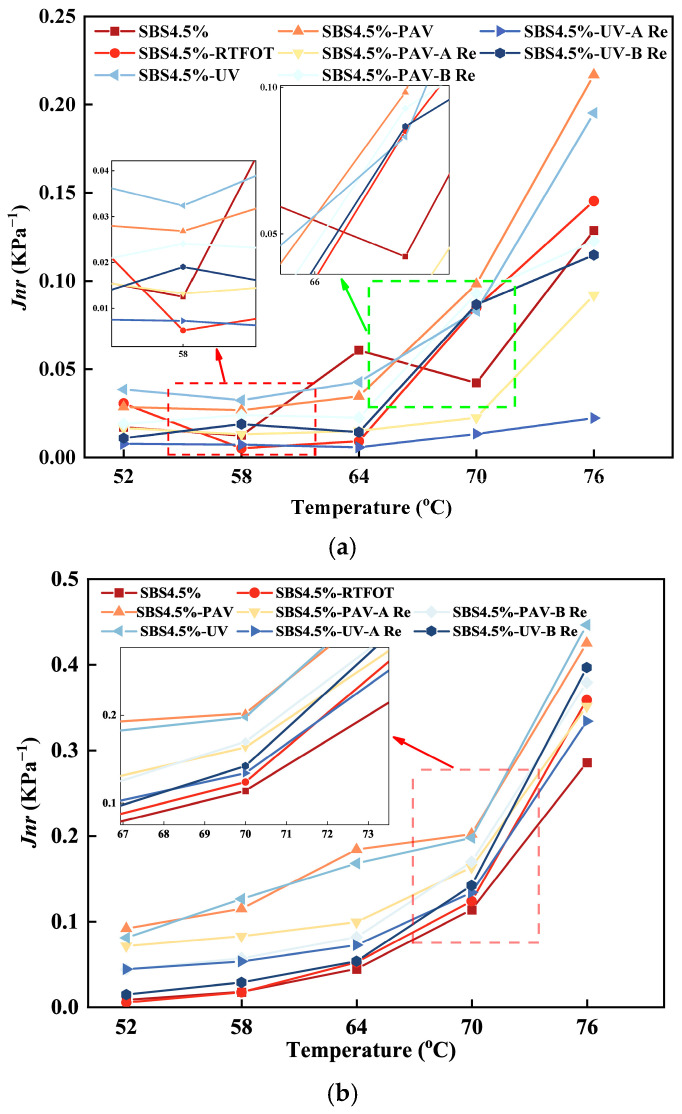
Variation in irrecoverable creep compliance with temperature for different binders under a compressive stress of (**a**) 0.1 kPa and (**b**) 3.2 kPa.

The unrecoverable creep compliance for different types of asphalt binder can be obtained by frequency scanning, as shown in Figure 7.

As shown in Figure 7, the original SBS asphalt binder consistently maintains the lowest *J_nr_* value under a stress level of 0.1 kPa, demonstrating optimal resistance to deformation. In contrast, aging significantly increases the material deformation susceptibility. Specifically, PAV aging causes the Jnr value to rise sharply to approximately 0.17 kPa^−1^, representing an increase of 340%, while UV aging has a relatively weaker effect, with the Jnr value increasing to about 0.1 kPa^−1^. Notably, when the stress level increases to 3.2 kPa, the *J_nr_* values of all samples show a clear upward trend, particularly for the PAV-aged sample, which further increases to about 0.38 kPa^−1^, indicating significant sensitivity to stress variations. This occurs because PAV aging forms a dense three-dimensional network through oxidative cross-linking, significantly restricting the molecular chain mobility, whereas UV aging mainly damages the surface polymer network, thus having a relatively limited impact on the overall fluidity [40]. In terms of rejuvenation effects, the two rejuvenators exhibit distinct differences. At 0.1 kPa stress, rejuvenator A restores the *J_nr_* value of UV-aged asphalt to approximately 0.05 kPa^−1^, nearly reaching the level of the original asphalt, while rejuvenator B shows relatively limited effectiveness, with the *J_nr_* value remaining at a higher level. More importantly, under the high stress condition of 3.2 kPa, the advantage of rejuvenator A becomes more pronounced, as its *J_nr_* value is significantly lower than that of the rejuvenator B group. Rejuvenator A, with its high vegetable oil content, effectively replenishes the light components and significantly improves the molecular chain mobility, thereby reducing the tendency for irreversible deformation. In contrast, rejuvenator B focuses on reconstructing the elastic network through SBS polymer and restorative agents. Although this approach effectively enhances stiffness, it also somewhat restricts the free movement of molecular chains, resulting in maintained higher deformation sensitivity.

#### 3.1.3. Analysis of BBR Results

(1) Creep stiffness modulus.

**Figure 8:**
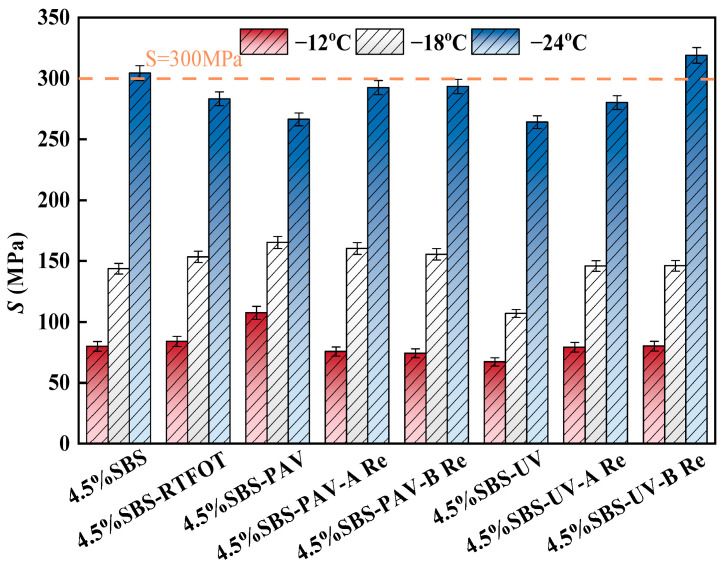
Variation in creep stiffness modules for different binders with different temperatures.

The creep stiffness modulus of different types of asphalt binder can be obtained from the BBR, as shown in Figure 8.

As shown in Figure 8, the original SBS asphalt binder maintains the lowest creep stiffness values under the relatively high temperature (−12 °C and −18 °C), while the *S* value shows a slight increase but remains at relatively low levels after RTFOT short-term aging. In contrast, PAV aging leads to a significant increase in the *S* value. However, the creep stiffness modules will be decreased under PAV and UV aging for the extremely low temperature (−24 °C). In terms of rejuvenation effects, for PAV-aged and UV-aged asphalt, rejuvenator A demonstrates good restoration performance, bringing the *S* value back to a level close to that of the original asphalt binder. In comparison, rejuvenator B shows better effectiveness, with the S-value remaining significantly higher than that of the rejuvenator A group. It is noteworthy that for PAV-aged asphalt, both rejuvenators effectively increase the *S* value, with rejuvenator B exhibiting a relative advantage, particularly under the extreme low-temperature condition of −24 °C. This difference occurs primarily because rejuvenator A, with its high vegetable oil content, effectively replenishes the light components and significantly restores the molecular chain mobility, thereby improving the low-temperature flexibility. In contrast, rejuvenator B mainly repairs aging damage by reconstructing the elastic network, thus maintaining higher stiffness while enhancing the low-temperature performance [41].

(2) Creep rate.

**Figure 9:**
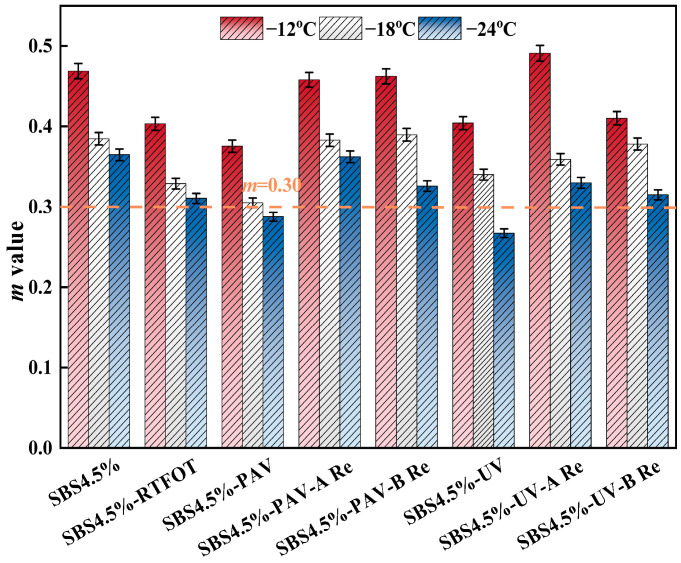
Variation in creep rate for different binders with different temperatures.

The creep rate of different types of asphalt binder can be obtained from the BBR test, as shown in Figure 9.

As shown in Figure 9, the original SBS asphalt binder maintains the highest *m* value across all three test temperatures, while after RTFOT aging, the *m* value decreases slightly but remains relatively high. In contrast, UV aging causes a noticeable reduction in the *m* value, particularly at −24 °C, where it drops to approximately 0.27, indicating that aging significantly weakens the material stress relaxation capacity. Notably, PAV aging leads to a further substantial decrease in the *m* value, which falls to about 0.18 at −24 °C, representing a reduction of over 50%. This demonstrates that long-term aging severely compromises the stress relaxation capability. The difference occurs because UV aging degrades the SBS polymer network through photo-oxidation, reducing the flexibility and mobility of molecular chains, while PAV aging forms a rigid network through more intensive molecular chain cross-linking and asphalt aggregation, further restricting the molecular chain mobility and significantly lowering the stress relaxation rate [42]. Regarding the rejuvenation effects, rejuvenator A demonstrates excellent restoration performance for UV-aged asphalt, bringing its *m* value close to the original level. However, rejuvenator B shows weaker effectiveness for UV aging, with the *m* value remaining significantly lower than that of the rejuvenator A group. In comparison, for PAV-aged asphalt, both rejuvenators effectively increase the m-value, with rejuvenator B exhibiting a relative advantage, particularly under the extreme low-temperature condition of −24 °C. This difference in restoration effectiveness arises because rejuvenator A, with its high vegetable oil content, effectively replenishes the light components and significantly improves the molecular chain mobility, making it particularly suitable for repairing polymer degradation caused by UV aging. In contrast, rejuvenator B primarily repairs aging damage by reconstructing the elastic network, a mechanism that proves more effective for the deeply cross-linked network formed during PAV aging [43].

### 3.2. Microscopic Characteristics of Different Aged and Rejuvenated Asphalt Binders

#### 3.2.1. Analysis of FTIR Test Results

**Figure 10:**
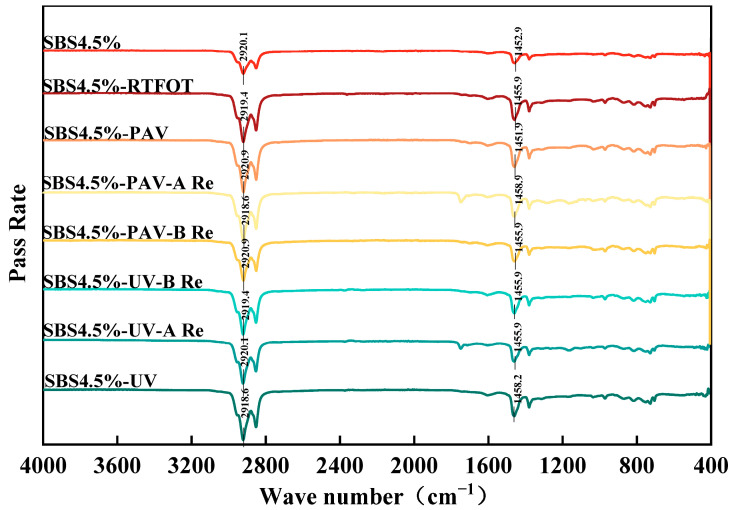
Comparison of infrared spectra of different types of asphalt binder.

FTIR can be used to obtain the corresponding wavenumbers of infrared spectra for different types of binder, as shown in Figure 10.

As shown in Figure 10, the infrared spectra of original, aged, and rejuvenated SBS modified asphalt binders exhibits distinct variations in characteristic absorption peaks, reflecting the molecular structure changes induced by aging and rejuvenation. The strong absorption bands at 2918 cm^−1^ and 2851 cm^−1^ persist across all samples, indicating that the aliphatic backbone structure of the asphalt remains largely intact. The significant enhancement of the characteristic peaks at 1598 cm^−1^, 1456 cm^−1^, and 1376 cm^−1^ in the rejuvenated samples demonstrates the effective restoration of aromatic components lost during oxidative aging.

Notably, two rejuvenators show distinct mechanistic differences. Rejuvenator A, primarily composed of oil fractions, mainly functions by supplementing light oil components to physically dilute aging products, as evidenced by the noticeable reduction in the sulfoxide group characteristic peak intensity at 1031 cm^−1^. In contrast, rejuvenator B not only interacts with oxidized species through its active components but also partially repairs the damaged SBS network, as confirmed by the enhanced peaks at 811 cm^−1^ and 721 cm^−1^ corresponding to aromatic ring substitutions [44].

Particularly insightful is the relatively stable intensity of the ester carbonyl characteristic peak at 1739 cm^−1^ across all rejuvenated samples, providing crucial evidence for understanding the two rejuvenators’ mechanisms. The research reveals that while the oil fractions in rejuvenator A are primarily dispersed in the aged asphalt through physical blending, and the active components in rejuvenator B specifically interact with the SBS network, neither induces the chemical conversion of ester carbonyl groups. These molecular-level findings confirm that both rejuvenators achieve performance recovery principally through physical means. Rejuvenator A focuses on replenishing lost light components to restore the system’s compositional balance, whereas rejuvenator B not only supplements the components but also conducts targeted repair of the damaged polymer network through its unique chemical composition. This essentially constitutes structural reconstruction at the physical level, rather than the chemical reversal of the aging process.

**Figure 11:**
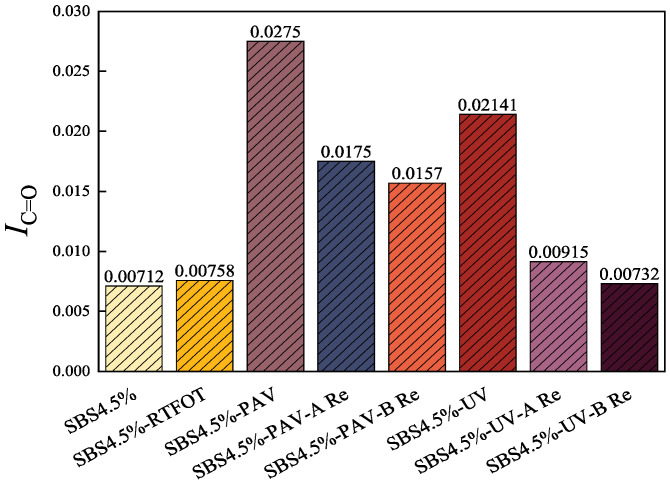
Carbonyl index of different asphalt binders.

**Figure 12:**
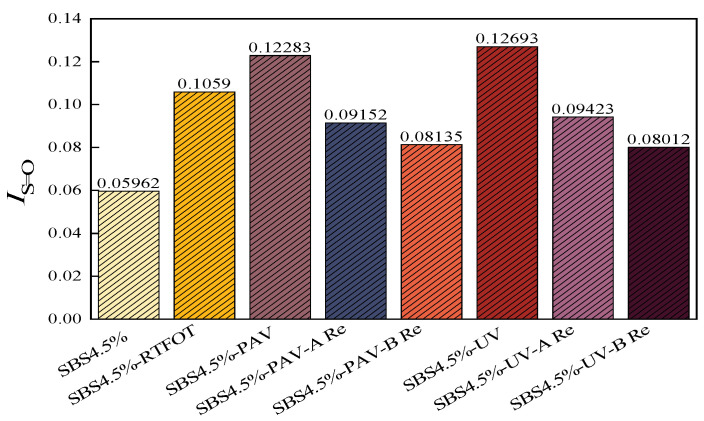
Sulfoxide index of different asphalt binders.

To further elucidate the microstructural evolution of SBS modified asphalt binders under different aging levels and rejuvenation treatments, FTIR was employed to quantitatively analyze the key chemical functional group indices characterizing the degree of oxidation. To bridge the gap between physical distribution and chemical evolution, the carbonyl and sulfoxide indexes were analyzed as shown in Figure 11 and Figure 12.

The indices demonstrate a sharp increase in polar functional groups during aging, with UV aging exhibiting the highest oxidation levels. This chemical enrichment of polar groups explains the formation of the polar aggregates that were previously identified as LMS in the GPC analysis.

Upon rejuvenation, rejuvenator B demonstrated a superior capacity for chemical mitigation, reducing the carbonyl and sulfoxide indices more aggressively than rejuvenator A. For instance, in UV-aged samples, rejuvenator B brought the *I*_C=O_ down to 0.00732, a value nearly equivalent to the original binder, whereas rejuvenator A only reached 0.00915. This confirms that while both agents function primarily through physical means, rejuvenator B provides a more potent chemical dilution effect. The higher *I*_S=O_ and *I*_C=O_ levels remaining in the rejuvenated binder after the work of rejuvenator A, coupled with its more balanced LMS/SMS ratio, suggest that rejuvenator A focuses on structural equilibrium without over-diluting the chemical backbone of the binder.

#### 3.2.2. Analysis of GPC Test Results

**Figure 13:**
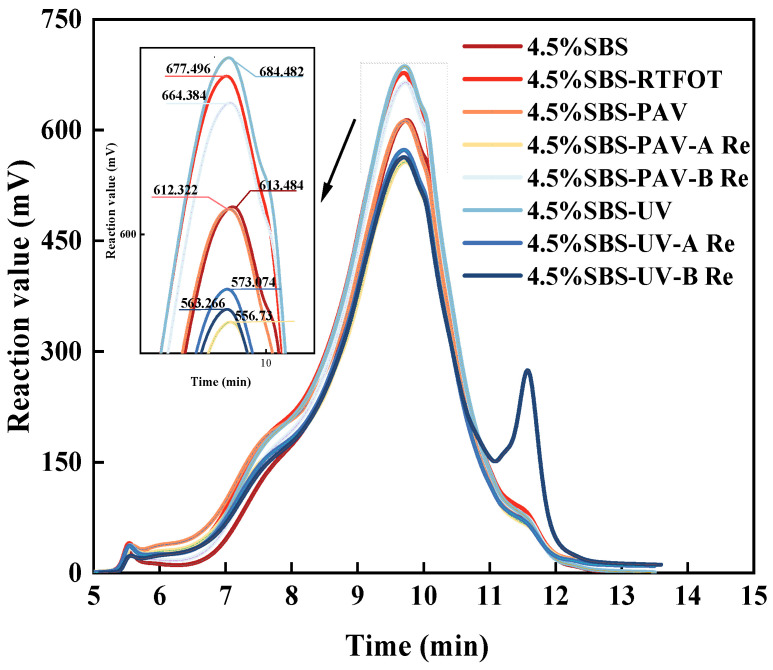
Variation in reaction value with time for different type of asphalt binder.

**Figure 14:**
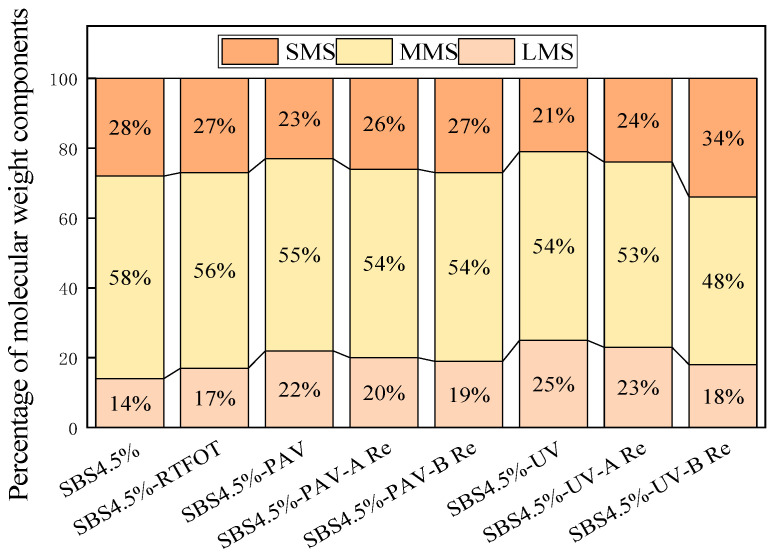
Evolution of molecular weight distribution for SBS modified asphalt binders.

GPC is to separate the molecular weight by the different residence times caused by the different size particles in the column. The higher molecular weight polymer has a short residence time in the column, and the lower molecular weight polymer has a long residence time. The molecular weight size and molecular weight distribution can be obtained. The test results are shown in Figure 13. To further quantify the MWD evolution, the proportions of LMS, MMS, and SMS were calculated in Figure 14.

As shown in Figure 13, the GPC analysis of the SBS modified asphalt binder reveals typical polymer distribution features. A prominent peak at 5 to 6 min corresponds to the high-molecular-weight SBS component, which elutes early due to its large hydrodynamic volume. Generally, high-molecular-weight species contribute to high-temperature stability, while lower molecular weight fractions are essential for maintaining low-temperature ductility and proper viscosity. Compared with the unaged binder, the GPC curves of aged asphalt binders exhibit a distinct leftward shift, indicating an overall increase in molecular weight. This phenomenon arises from aging-induced fragmentation, oxidative cross-linking, and the migration of light components to heavier fractions, which collectively enhance high-temperature stiffness but diminish low-temperature crack resistance, as observed macroscopically [45].

As shown in Figure 14, the quantitative results demonstrate a clear transition from SMS to LMS during the aging process. Notably, UV aging resulted in the highest LMS concentration (25%), representing a 78.5% relative increase compared to the original binder. This quantitative surge in LMS corroborates the leftward shift in the GPC curves and underscores the severity of photo-oxidative aging in transforming light fractions into polar aggregates.

Upon rejuvenation, a restorative trend is observed in both the GPC curves and the component proportions. Asphalt treated with rejuvenator A exhibits a significant rightward shift in its GPC curve, reflecting the effective replenishment of lost light components. Figure 11 further reveals that rejuvenator A produces a molecular profile that more closely mirrors the original state (SBS 4.5%), suggesting a more balanced chemical restoration. In contrast, while rejuvenator B induces a more aggressive reduction in LMS and a substantial boost in SMS (reaching 34% for the UV-B regenerated sample), its distribution deviates significantly from the original binder. This suggests that rejuvenator B may act through a powerful solvent effect or “over-dilution” with light oils, whereas the structural equilibrium achieved by rejuvenator A is likely the underlying reason for the superior recovery of viscoelastic properties observed in the macroscopic performance test.

This molecular redistribution is further supported by FTIR analysis, which confirms that the rejuvenators effectively restore the aromatic structures and light components. After rejuvenation, the characteristic peaks at 1456 cm^−1^ and 1376 cm^−1^ are significantly increased, while the oxidative sulfoxide peak at 1031 cm^−1^ is decreased. Correspondingly, the polydispersity index is reduced as the molecular weights shift back to lower ranges. Ultimately, the combined FTIR and GPC analyses confirm that the rejuvenators, particularly rejuvenator A, not only dilute oxidative byproducts but also partially restore the original binder structures, leading to a comprehensive improvement in both the chemical and viscoelastic properties.

## 4. Conclusions

Three kinds of aged asphalt binder were prepared in this study to effectively restore the performance of aged SBS modified asphalt binder. The rheological performance test and microscopic characteristic analysis were used to systematically analyze the effects of regeneration agents on the macroscopic properties and microstructure of aged asphalt binder. The regeneration mechanisms of the two regeneration agents on thermal oxygen aging and ultraviolet-aged asphalt binder were compared and analyzed. The main findings are summarized as follows:

(1) PAV aging induces more severe stiffening in SBS modified asphalt than UV aging, due to oxidative cross-linking. For high-temperature performance, rejuvenator A (oil-rich) more effectively restores the modulus and rutting resistance of UV-aged asphalt, while rejuvenator B (polymer-containing) better repairs the cross-linked network of PAV-aged asphalt, demonstrating selective restoration based on the aging mechanism.

(2) PAV aging more severely degrades the elastic recovery and deformation resistance of SBS modified asphalt than UV aging, particularly under high stress. Two rejuvenators significantly improve the rheological properties of the aged asphalt binder. Oil-rich rejuvenator A optimally restores UV-aged asphalt, while polymer-based rejuvenator B shows limited recovery for PAV-aged samples due to its network-rebuilding mechanism restricting molecular mobility.

(3) PAV and UV aging will lead to a decrease in creep stiffness modulus and *m*-value at extremely low temperature, while the addition of rejuvenators will restore their low-temperature performance. Comparatively, both rejuvenators have comparable recovery effects on PAV aging, while rejuvenator A shows better recovery of the *S* value, while rejuvenator B also demonstrates good recovery of the *m*-value.

(4) FTIR analysis reveals that aging significantly increases carbonyl and sulfoxide indices of SBS modified asphalt binder, especially after PAV and UV aging. Rejuvenator B exhibits superior chemical dilution, reducing these indices nearly to original levels, whereas rejuvenator A shows a modest reduction, with the higher *I*_*S*=*O*_ and *I*_*C*=*O*_ levels remaining in the rejuvenated binder.

(5) GPC analysis demonstrates an aging-induced molecular weight increase and LMS formation. Rejuvenator A can reduce the LMS formed after aging to some extent, but its recovery effect is quite limited (reducing LMS by 2%). Conversely, rejuvenator B aggressively reduces the LMS but causes over-dilution, yielding molecular distributions deviating from the original binder.

This study was mainly conducted using the simulated aging conditions, and the investigation related to the rejuvenation of reclaimed asphalt pavement (RAP) will be further conducted to enhance the value of the developed rejuvenators.

## Figures and Tables

**Table 1:** Technical indicators of base asphalt binder.

Indicators		Unit	Measured Values	Specification Requirements
Penetration (25 °C, 100 g, 5 s)		0.1 mm	87.2	80~100
Ductility (5 cm/min, 10 °C)		cm	>100	≥100
Softening point		°C	47.4	≥42
After RTFOT (85 min)	Penetration ratio	%	66	≥54
	Loss of mass	%	0.06	−0.8~0.8
	Ductility	cm	9.1	≥6

**Table 2:** Material compositions of rejuvenator A.

Material Compositions	Percentage (%)
Waste vegetable oil	74
Plasticizer	15
Toughening agent	4
Tackifier	7

**Table 3:** Material compositions of rejuvenator B.

Material Compositions	Percentage (%)
Softener	50
Plasticizer	20
Polymerization agent	10
Aluminate coupling agent	3
Restorative	10
Antioxidant BHT	2
UV absorber UV-326	2
SBS	3

## Data Availability

The original contributions presented in this study are included in the article. Further inquiries can be directed to the corresponding authors.

## References

[1] Ma Q., He H., Yu W., Xu J., Xu Q., Xue J., Jin Y., Zhu R., Han C., Wang Z. (2025). Novel rejuvenators for synergistically improving the high/low-temperature performance of epoxy-rejuvenated styrene-butadiene-styrene (SBS) modified bitumen. Constr. Build. Mater..

[2] Wen Y., Ma F., Fu Z., Li C., Dai J., Dong W., Shi K., Zhu C. (2024). Evaluation on the fatigue and self-healing properties of aged and rejuvenated SBS-modified asphalt. Constr. Build. Mater..

[3] Cavalli C.M., Wu W., Poulakis L. (2024). Bio-based rejuvenators in asphalt pavement: A comprehensive review and analytical study. J. Road Eng..

[4] Dai M., Hou J., Wei W. (2023). Research progress on aging and regeneration of SBS-modified asphalt. Value Eng..

[5] Zhang D., Zheng Y., Yuan G., Guo H., Zhou Q., Qian G., Liang B. (2023). Comparative analysis of rheological and microscopic performance of SBS modified asphalt based on field aging and laboratory aging. Fuel.

[6] Shi K., Ma F., Liu J., Fu Z., Song R., Yuan D., Ogbon A.W. (2024). Evolution of SBS-modified asphalt performance under aging and rejuvenation cycle conditions. Constr. Build. Mater..

[7] Xiao J., Ding X., Yu J., Xiao H., Liu Q. (2024). Analysis of the aging and regeneration mechanism of asphalt and the influencing factors of the utility of the regeneration agent. Transp. Sci. Technol. Manag..

[8] Li B., Han J., Wei D., Ji H., Yao T., Wang H., Han J., Zhang Y. (2024). A molecular dynamics simulation study on the recovery performance of aged asphalt binders by waste vegetable oil rejuvenators. J. Clean. Prod..

[9] Song J., He L., Wang X., Luo L., Li W. (2017). Microscopic aging mechanism of SBS-modified asphalt in RTFOT. J. Highw. Transp. Res. Dev..

[10] Zhou Y., Li Q., Tong Y., Qu F., Wen G., Wu C. (2022). Effect of regeneration agent on macroscopic properties and microstructure of SBS-modified asphalt. Highway.

[11] Cao Z., Chen M., Liu Z., He B., Yu J., Xue L. (2019). Effect of different rejuvenators on the rheological properties of aged SBS modified bitumen in long term aging. Constr. Build. Mater..

[12] Han M., Li Z., Zhao Z., Zhao H. (2016). Research progress on aging and anti-aging of SBS-modified asphalt. Sci. Technol. Rev..

[13] Sun J., Yu S., Xu N., Li N. (2024). Evaluation of asphalt-aggregate interface adhesion considering aging. J. Highw. Transp. Sci. Technol..

[14] Wang Y., Sun L., Qin Y. (2015). Aging mechanism of SBS modified asphalt based on chemical reaction kinetics. Constr. Build. Mater..

[15] Liu C., Du J., Wu C., Liu K., Jiang K. (2022). Low-temperature crack resistance of wood tar-based rejuvenated asphalt based on viscoelastic rheological method. Int. J. Pavement Res. Technol..

[16] Zhang Y., Zhang X., Xu M., Zou G., Yu J. (2024). A review of research on coupled aging of asphalt binders. J. Highw. Transp. Sci. Technol..

[17] Yu J., Lin Z., Zou G., Yu H., Leng Z., Zhang Y. (2024). Long-term performance of recycled asphalt mixtures containing high RAP and RAS. J. Road Eng..

[18] Zhu C., Xu Z., Zhang H., Wang Z., Li D., Su N., Zhang D. (2025). Rheological and micro characteristics of asphalt containing different nanocomposite ratios of CeO2/MMT during thermal-oxidative and ultraviolet aging. Constr. Build. Mater..

[19] Ju Z., Ge D., Lv S., Liu Q., Wang X., Bai Y. (2024). Rheological and microscopic characterization and correlation analysis of asphalt under high-intensity ultraviolet radiation. Case Stud. Constr. Mater..

[20] Li B., Han J., Nan X., Li X., Li X., Zhang P. (2023). Adhesion characteristics and spectroscopic analysis of regenerated ultraviolet aged asphalt binder using waste vegetable oil. Case Stud. Constr. Mater..

[21] Zhao Y., Gu F., Huang X. (2011). Analysis of aging characteristics of SBS-modified asphalt based on FTIR. J. Build. Mater..

[22] (2011). Standard Test Methods of Bitumen and Bituminous Mixtures for Highway Engineering.

[23] Ābele A., Merijs-Meri R., Bērziņa R., Zicāns J., Haritonovs V., Ivanova T. (2021). Effect of bio-oil on rheological and calorimetric properties of RTFOT aged bituminous compositions. Int. J. Pavement Res. Technol..

[24] Eltwati A., Mohamed A., Hainin M.R., Jusli E., Enieb M. (2024). Rejuvenation of aged asphalt binders by waste engine oil and SBS blend: Physical, chemical, and rheological properties of binders and mechanical evaluations of mixtures. Constr. Build. Mater..

[25] Jin T., Feng Y., Li M., Liu L., Yuan J., Sun L. (2024). Laboratory short-term aging of crumb rubber modified asphalt: RTFOT temperature optimization and performance investigation. J. Clean. Prod..

[26] (2017). Standard Practice for Accelerated Weathering Test Conditions and Procedures for Bituminous Materials (Fluorescent UV, Water Spray, and Condensation Method).

[27] (2015). Standard Test Method for Determining the Rheological Properties of Asphalt Binder Using a Dynamic Shear Rheometer.

[28] (2019). Standard Method of Test for Multiple Stress Creep Recovery (MSCR) Test of Asphalt Binder.

[29] (2016). Standard Test Method for Determining the Flexural Creep Stiffness of Asphalt Binder Using the Bending Beam Rheometer (BBR).

[30] (2013). Standard Practice for General Techniques for Obtaining Infrared Spectra for Qualitative Analysis.

[31] Ren S., Liu X., van Aggelen M., Lin P., Erkens S. (2024). Do different chemical and rheological properties act as effective and critical indicators for efficiency evaluation of rejuvenated bitumen?. Constr. Build. Mater..

[32] Ma J., Sun G., Sun D., Yu F., Hu M., Lu T. (2021). Application of gel permeation chromatography technology in asphalt materials: A review. Constr. Build. Mater..

[33] Xu S., Zhao Z., Zhao Y., Wan C., Wang X. (2026). Multi-scale characterization of styrene–butadiene–styrene modified bitumen during thermal aging. Mater. Struct..

[34] (2013). Standard Practice for Molecular Weight Averages and Molecular Weight Distribution of Hydrocarbon, Rosin and Terpene Resins by GPC.

[35] Xiao G., Wei Y. (2021). Effects of amorphous poly alpha olefin (APAO) and polyphosphoric acid (PPA) on the rheological properties, compatibility, and stability of asphalt binder. Materials.

[36] Yan Z., Zhang Q. (2023). Study on the performance of bio-oil regenerator heat regeneration aging SBS-modified asphalt and mixture. New Build. Mater..

[37] Ding H., Xu D. (2024). Rheological analysis of multiple stress creep and recovery (MSCR) test. J. Highw. Transp. Res. Dev..

[38] Dong Z., Yuan M. (2023). Operative analysis of rheological and microscopic performance of SBS-modified asphalt based on field aging and laboratory aging. Fuel.

[39] Sun Y., Lv B., Gong H., Xu H., Wang W., Xu B., Chen J. (2023). Approach for accurately characterizing nonlinear viscoelastic and nonrecoverable properties of asphalt binders based on MSCR test. Constr. Build. Mater..

[40] Han X., Niu X., Zhang Z., Cao Z., Wang R., Feng Z., Yu J. (2025). Laboratory evaluation of ultraviolet aging performance of regenerated SBS modified bitumen based on active flexible rejuvenators with different molecular structures. Constr. Build. Mater..

[41] Cannone Falchetto A., Moon K.H., Wang D., Riccardi C. (2018). Investigation on the cooling medium effect in the characterization of asphalt binder with the bending beam rheometer. Can. J. Civ. Eng..

[42] Ren H., Qian Z., Huang W., Li H., Liu Y. (2022). Low-temperature thermal cracking performance of waterborne epoxy asphalt emulsion mastic based on bending beam rheometer (BBR). Constr. Build. Mater..

[43] Huang M., Wu W., Gao J., Chen B., Li Z. (2025). Effects of lignin compositions on the antioxidative and rheological properties of asphalt. Constr. Build. Mater..

[44] Madeira N.C.L., de Souza L.M., Pereira A.R., Chinelatto L.S., Cravo M.C., Nascimento L.A.H.D., Lacerda V., Romão W. (2024). Study of thermal and photochemical aging of saturates, naphtenic-aromatics, resins, and asphaltene fractions of asphalt cement by FTIR and FT-ICR MS. Fuel.

[45] Liu J., Zhang L., Cheng Y., Wang S. (2012). Molecular weight distribution of polystyrene analyzed by gel permeation chromatography and light scattering techniques. Polym. Test..

